# The female urinary microbiota in relation to the reproductive tract microbiota

**DOI:** 10.46471/gigabyte.9

**Published:** 2020-11-27

**Authors:** Chen Chen, Lilan Hao, Weixia Wei, Fei Li, Liju Song, Xiaowei Zhang, Juanjuan Dai, Zhuye Jie, Jiandong Li, Xiaolei Song, Zirong Wang, Zhe Zhang, Liping Zeng, Hui Du, Huiru Tang, Tao Zhang, Huanming Yang, Jian Wang, Susanne Brix, Karsten Kristiansen, Xun Xu, Ruifang Wu, Huijue Jia

**Affiliations:** ^1^ BGI-Shenzhen, Shenzhen 518083, China; ^2^ China National Genebank, BGI-Shenzhen, Shenzhen 518120, China; ^3^ Peking University Shenzhen Hospital, Shenzhen 518036, China; ^4^ Shenzhen Key Laboratory on Technology for Early Diagnosis of Major Gynecological diseases, Shenzhen, PR China; ^5^ Department of Biology, Ole MaalØes Vej 5, University of Copenhagen, Copenhagen, Denmark; ^6^ Shenzhen Key Laboratory of Human Commensal Microorganisms and Health Research, BGI-Shenzhen, Shenzhen, China; ^7^ James D. Watson Institute of Genome Sciences, Hangzhou, China; ^8^ Department of Biotechnology and Biomedicine, Technical University of Denmark, Soltofts Plads, Building 221, 2800 Kgs. Lyngby, Denmark; ^9^ Macau University of Science and Technology, Taipa, Macau 999078, China

## Abstract

Human urine is traditionally considered to be sterile, and whether the urine harbours distinct microbial communities has been a matter of debate. Potential links between female urine and reproductive tract microbial communities is currently not clear. Here, we collected urine samples from 147 Chinese women of reproductive age and explored the nature of colonization by 16S rRNA gene amplicon sequencing, quantitative real-time PCR, and live bacteria culture. To demonstrate the utility of this approach, the intra-individual Spearman’s correlation was used to explore the relationship between urine and multiple sites of the female reproductive tract. PERMANOVA was also performed to explore potential correlations between the lifestyle and various clinical factors and urinary bacterial communities. Our data demonstrated distinct bacterial communities in urine, indicative of a non-sterile environment. *Streptococcus*-dominated, *Lactobacillus*-dominated, and diverse type were the three most common urinary bacterial community types in the cohort. Detailed comparison of the urinary microbiota with multiple sites of the female reproductive tract microbiota demonstrated that the urinary microbiota were more similar to the microbiota in the cervix and uterine cavity than to those of the vagina in the same women. Our data demonstrate the potential connectivity among microbiota in the female urogenital system and provide insight and resources for exploring diseases of the urethra and genital tract.

## Data Description

### Purpose of data acquisition

The role of microbiota in the vaginal environment has received a lot of attention over the past decade, while the female upper reproductive tract was traditionally believed to be sterile and mostly studied in the context of infections or incontinence [1]. Despite continued controversy, the presence of microorganisms beyond the cervix (i.e. the female upper reproductive tract) is increasingly recognized even in non-infectious conditions [2]. Like the female upper reproductive tract, the sterile hypothesis of urine has also been overturned by emerging evidence that indicates the existence of microorganisms in the urinary tract by culturing or sequencing approaches [3, 4]. A recent study using an expanded quantitative urine culture in combination with whole-genome sequencing has isolated and sequenced the genomes of 149 bacterial strains from catheterized urine of both symptomatic and asymptomatic peri-menopausal women [5]. It also showed highly similar strains of commensal bacteria in both the bladder and vagina of the same individual [5]. Another study analysed the urinary microbiota of 189 individuals using 16S rRNA gene amplicon sequencing and suggested that the urethra and bladder can harbour microbial communities distinct from the vagina [6]. However, the relationship between female urine microbiota and the upper reproductive tract microbiota has so far not been studied.

Here, we present a dataset of the urinary microbiota for a relatively large cohort of 147 women of reproductive age. Together with our recently published study of peritoneal fluid, uterine, and vaginal samples from the same individuals [2], this data shows that although urinary microbiota contain larger populations of *Lactobacillus* and *Streptococcus*, they are more similar to the microbiota of the cervix and uterine cavity, in accordance with the anatomical opening of the bladder. Together with a wealth of metadata, we demonstrate that these data are useful for exploring the potential of the urinary microbiota for clinical diagnosis.

## Methods

**Figure 1.**
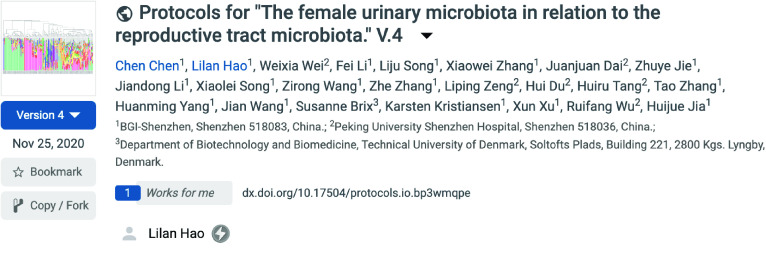
Protocol collection for sequencing and analysing female urinary microbiota. https://www.protocols.io/widgets/doi?uri=dx.doi.org/10.17504/protocols.io.bp3wmqpe

A protocol collection including methods for DNA extraction, bioinformatics analysis and quantitative real-time PCR is available via protocols.io (Figure 1) [7].

### Sample collection

In this study, a total of 147 reproductive age women (age 22–48) were recruited by Peking University Shenzhen Hospital [8]. All participants were reproductive age women who underwent hysteroscopy and/or laparoscopy for conditions without infections, such as hysteromyoma, adenomyosis, endometriosis, or salpingemphraxis. Subjects with other related diseases, such as vaginal inflammation, severe pelvic adhesion, endocrine or autoimmune disorders were removed. Pregnant women, breastfeeding women, and menstruating women at the time of sampling were also excluded. None of the subjects received any antibiotic treatments or vaginal medications within two weeks of sampling. In addition, no cervical treatment was performed within the previous 7 days, no vaginal douching was performed within 5 days, and no sexual activity was performed within at least 2 days.

137 self-sampling morning mid-stream urine samples were collected between December 2013 and July 2014 prior to the surgery (**sample_metadata.csv** [8]), and then stored at −80 ^°^C until they were transported on dry ice to BGI-Shenzhen for sequencing. The samples from an additional 10 women were collected for validation purposes by a doctor during the surgery in July 2017. For each operation, a urine catheter was inserted into the disinfected urethra to collect mid-stream urine. For each sample of urine collected through a catheter, an identical volume of saline solution was set as the control sample. The samples were then placed at 4 ^°^C, transported to BGI-Shenzhen, and processed within 6 hours. A portion of each sample was used for culturing live bacteria and the rest was used for sequencing.

### DNA extraction and 16S rRNA amplicon sequencing

Genomic DNA extraction was carried out following the protocol [9]. The primers 515F and 907R were utilized for PCR amplification of the hypervariable regions V4-V5 of the bacterial 16S rRNA gene. The 907R primer includes a unique barcoded fusion. The primer sequences were: 515F: 5^*′*^-GTGCCAGCMGCCGCGGTAA-3^*′*^ and 907R: 5^*′*^-CCGTCAATTCMTTTRAGT-3^*′*^, where M denotes A or C and R denotes purine. The conditions for PCR amplification were: 3 min of denaturation at 94 ^°^C, followed by 25 cycles of 45 s at 94 ^°^C (denaturing), 60 s at 50 ^°^C (annealing), and 90 s at 72 ^°^C (elongation), followed by a final elongation for 10 min at 72 ^°^C. The amplification products were purified by the AxyPrep™ Mag PCR Clean-Up Kit (Axygen, USA). The amplicon libraries were constructed with an Ion Plus Fragment Library Kit (Thermo Fisher Scientific Inc.) [10], then sequenced by the Ion PGM™ Sequencer with the Ion 318™ Chip v2 with a read length of 400 bp (Thermo Fisher Scientific Inc., Ion PGM™ Hi-Q™ OT2 Kit, Cat.No: A27739; Ion PGM™ Hi-Q™ Sequencing Kit, Cat.No: A25592) [11]. All experiments were performed in the laboratory of BGI-Shenzhen.

### Processing of sequencing reads

The raw sequencing reads were first subjected to Mothur (Mothur, RRID:SCR_011947; V1.33.3) [12] for filtering out the low-quality reads meeting the following criteria: (1) reads shorter than 200 bp; (2) reads not matching the degenerated PCR primers for up to two errors; (3) reads with an average quality score less than 25. A total of 8,812,607 reads, with an average of 57,225 reads per sample (a minimum of 1113 reads and a maximum of 194,564 reads) were obtained. Subsequently, the sequences with identity greater than 97% were clustered into Operational Taxonomic Units (OTUs) using the QIIME (QIIME, RRID:SCR_008249; V1.8.0) uclust programme [13], where each cluster was thought of as representing a species. The seed sequences of each OTU were aligned against the Greengene reference sequences (gg_13_8_otus) for annotation using Mothur. The detailed analysis workflow was deposited in protocols.io [14].

We also calculated the Unifrac distance using QIIME based on taxonomic abundance profiles at the OTU level [11].

### PERMANOVA on the influence of phenotypes

Permutational multivariate analysis of variance (PERMANOVA) was used to assess the effect of different covariates based on the relative abundances of OTUs of the samples [15, 16] using Bray-Curtis and UniFrac distance and 9999 permutations from the vegan package (vegan, RRID:SCR_011950) in R [16, 17].

### Quantitative real-time PCR

We quantified the four *Lactobacillus* species, including *L.iners*, *L jensenii*, *L. crispatus* and *L. gasseri* using the modified qPCR protocol [18]. SYBR Premix Ex Taq GC (TAKARA) was used and the reactions were run on a StepOnePlus Real-time PCR System (Life Technologies). Each PCR reaction mixture contained 10 μl of 2×SYBR Premix Ex Taq GC, 0.2 μM forward primer, 0.2 μM reverse primer, 1.6 μl of DNA sample, and 8.2 μl of ultrapure water to make up the final reaction volume of 20 μl. Each run included a standard curve and all samples were amplified in triplicate. Ultrapure water was used as the blank control template.

To construct the standard curves, the sequencing-confirmed plasmids of four species were used after quantification with a Qubit Fluorometer and serial 10-fold dilutions. The amplification efficiency was (100 ±10)% and linearity values were all ≥0.99. The detailed procedure was deposited in protocols.io [19].

### Bacterial culturing

The urine samples and controls from 10 additional subjects were cultivated in the laboratory by spreading 100 μl of sample on different agars containing 5% horse blood, such as PYG agar (DSMZ 104 medium), BHI agar, and EG agar. The plates were incubated in both aerobic and anaerobic conditions at 37 ^°^C for 72 hours. To keep the medium anaerobically during culture, resazurin and cysteine-HCl were added as reducing agents. The genomic DNA of the isolates was extracted by the Bacterial DNA Kit (OMEGA) and then underwent 16S rRNA gene amplification using the universal primers 27F/1492R [20]. The amplicons were purified and sent for Sanger sequencing. The generated sequences were then submitted to BLAST on the EzBioCloud [21] for identification.

## Preliminary analysis and validation

### Microbiota composition of the urine

**Table 1:** Sequencing and annotation of the 137 samples from the exploratory cohort.

Sample name	Sequencing amount			% of reads annotated to taxa		Archive accession number
	#raw reads	#clean reads	#filtered reads	Genus	Species	
C001UR	52506	11326	9347	100.00%	75.51%	SAMEA5042945
C002UR	55955	14529	5930	100.00%	65.36%	SAMEA5042987
C003UR	61367	21181	16831	100.00%	91.06%	SAMEA5043040
C004UR	54585	18506	3325	100.00%	41.59%	SAMEA5042979
C005UR	52177	22856	20683	100.00%	92.62%	SAMEA5043003
C007UR	50766	15468	8737	100.00%	71.10%	SAMEA5043001
C008UR	53748	14062	6169	100.00%	64.05%	SAMEA5043004
C009UR	53383	12247	11327	100.00%	96.97%	SAMEA5043046
C011UR	47814	13058	11292	100.00%	66.76%	SAMEA5042941
C012UR	55279	16923	7484	100.00%	55.46%	SAMEA5042938
C014UR	55713	15175	8818	100.00%	64.31%	SAMEA5043060
C016UR	73372	22054	17669	100.00%	57.04%	SAMEA5043009
C018UR	69142	26505	23581	100.00%	12.12%	SAMEA5043006
C019UR	72249	17868	14440	100.00%	44.63%	SAMEA5043054
C020UR	54574	19391	5452	100.00%	63.83%	SAMEA5042942
C021UR	58118	17294	12123	100.00%	55.33%	SAMEA5042998
C023UR	47476	18452	16795	100.00%	16.09%	SAMEA5042947
C026UR	46583	16741	3267	100.00%	83.96%	SAMEA5042969
C028UR	88245	26955	19268	100.00%	19.93%	SAMEA5042984
C033UR	90431	31998	26496	100.00%	66.99%	SAMEA5043062
C035UR	63773	27044	24115	100.00%	97.31%	SAMEA5043037
C038UR	55562	10165	9208	100.00%	84.56%	SAMEA5042972
C039UR	77957	18891	15748	100.00%	33.38%	SAMEA5042963
C040UR	58940	12555	5438	100.00%	69.49%	SAMEA5043021
C041UR	60028	15361	9366	100.00%	78.38%	SAMEA5043000
C042UR	74086	14402	11088	100.00%	67.53%	SAMEA5042955
C043UR	74146	23691	18730	100.00%	60.19%	SAMEA5043032
C045UR	61249	17801	10367	100.00%	2.96%	SAMEA5043048
C047UR	47742	11940	3506	100.00%	54.25%	SAMEA5043024
C048UR	35550	1100	816	100.00%	62.75%	SAMEA5042931
C050UR	51565	18902	290	100.00%	72.76%	SAMEA5042936
C051UR	58783	10403	8234	100.00%	50.77%	SAMEA5042983
C053UR	32311	1653	26	100.00%	73.08%	SAMEA5043035
C055UR	45054	13326	6184	100.00%	56.40%	SAMEA5043016
C056UR	69173	24652	8282	100.00%	86.78%	SAMEA5043023
C057UR	64417	27033	24444	100.00%	98.11%	SAMEA5043059
C058UR	42089	1415	912	100.00%	4.28%	SAMEA5042935
C059UR	53642	12618	577	100.00%	74.70%	SAMEA5042930
C060UR	73930	22110	19192	100.00%	20.17%	SAMEA5043008
C062UR	63220	19932	17112	100.00%	79.58%	SAMEA5043012
C063UR	44974	1201	790	100.00%	53.42%	SAMEA5043039
C064UR	63505	15051	7134	100.00%	82.38%	SAMEA5042981
C065UR	53884	15094	13794	100.00%	52.97%	SAMEA5043027
C066UR	63269	16157	12090	100.00%	45.86%	SAMEA5042985
C067UR	55812	19047	2481	100.00%	86.86%	SAMEA5042986
C068UR	54396	17456	15352	100.00%	86.72%	SAMEA5042937
T000UR	57607	11995	9166	100.00%	37.51%	SAMEA5043045
T001UR	47924	13474	2849	100.00%	48.58%	SAMEA5043014
T002UR	63839	18381	3623	100.00%	49.71%	SAMEA5042975
T003UR	70242	19166	5255	100.00%	51.67%	SAMEA5042988
T004UR	67280	20578	2947	100.00%	57.24%	SAMEA5042943
T005UR	52820	12868	4931	100.00%	57.92%	SAMEA5043019
T006UR	79409	19472	13710	100.00%	19.58%	SAMEA5043017
T007UR	34173	1403	797	100.00%	50.06%	SAMEA5042999
T008UR	30074	1346	1044	100.00%	84.77%	SAMEA5043031
T009UR	58440	10386	7936	100.00%	81.92%	SAMEA5042950
T010UR	65382	17191	9801	100.00%	47.54%	SAMEA5042967
T011UR	38550	1163	464	100.00%	65.52%	SAMEA5043061
T012UR	75848	21956	4605	100.00%	52.62%	SAMEA5043049
T013UR	26872	1383	203	100.00%	74.38%	SAMEA5042996
T014UR	23298	1741	518	100.00%	93.44%	SAMEA5043042
T015UR	40653	2052	1470	100.00%	29.25%	SAMEA5042953
T016UR	58448	16261	3993	100.00%	58.75%	SAMEA5043053
T017UR	58703	19270	1929	100.00%	54.12%	SAMEA5042990
T018UR	54726	12668	7152	100.00%	17.18%	SAMEA5042970
T019UR	67711	14153	1606	100.00%	58.90%	SAMEA5042954
T020UR	89936	22579	17919	100.00%	68.74%	SAMEA5043052
T021UR	66094	14761	5276	100.00%	29.66%	SAMEA5042951
T022UR	28712	803	422	100.00%	74.88%	SAMEA5042940
T023UR	27738	1338	385	100.00%	30.65%	SAMEA5042961
T024UR	19345	824	55	100.00%	90.91%	SAMEA5042948
T025UR	29739	1578	1214	100.00%	76.85%	SAMEA5042995
T026UR	79923	17606	6269	100.00%	37.09%	SAMEA5042959
T027UR	61145	10093	6169	100.00%	59.51%	SAMEA5042949
T028UR	71755	19529	16118	100.00%	7.61%	SAMEA5042965
T029UR	58776	11505	8300	100.00%	6.04%	SAMEA5043018
T030UR	57098	7000	5119	100.00%	70.23%	SAMEA5042966
T031UR	49283	16113	1636	100.00%	38.63%	SAMEA5043063
T032UR	46822	15041	1187	100.00%	53.24%	SAMEA5042927
T033UR	63044	14272	10097	100.00%	80.89%	SAMEA5043043
T035UR	50618	12403	1122	100.00%	76.20%	SAMEA5043002
T036UR	78781	22492	17075	100.00%	84.36%	SAMEA5042982
T038UR	73752	15237	11784	100.00%	1.14%	SAMEA5043022
T039UR	58904	22286	19836	100.00%	95.82%	SAMEA5043015
T040UR	77039	15332	8059	100.00%	40.76%	SAMEA5043028
T041UR	58382	13735	11893	100.00%	97.26%	SAMEA5043056
T042UR	53948	17112	1392	100.00%	29.74%	SAMEA5043026
T043UR	72662	15446	10584	100.00%	16.18%	SAMEA5042956
T044UR	59818	19779	724	100.00%	73.48%	SAMEA5042993
T045UR	63627	21438	19282	100.00%	39.34%	SAMEA5043033
T046UR	58142	20606	912	100.00%	56.69%	SAMEA5042991
T047UR	24190	736	26	100.00%	73.08%	SAMEA5042978
T048UR	10255	441	53	100.00%	79.25%	SAMEA5043036
T049UR	63640	22520	20236	100.00%	98.73%	SAMEA5043010
T051UR	22322	1066	101	100.00%	77.23%	SAMEA5042997
T052UR	57909	11757	6419	100.00%	67.85%	SAMEA5043058
T053UR	63637	24574	21178	100.00%	99.17%	SAMEA5043034
T054UR	59194	18986	17148	100.00%	15.87%	SAMEA5042929
T055UR	70744	14983	2724	100.00%	84.07%	SAMEA5042962
T056UR	58876	19486	846	100.00%	65.84%	SAMEA5043051
T057UR	53598	13889	12456	100.00%	96.76%	SAMEA5042939
T058UR	18302	739	45	100.00%	64.44%	SAMEA5043044
T059UR	59974	15542	12378	100.00%	23.53%	SAMEA5042964
T060UR	21736	500	203	100.00%	89.16%	SAMEA5042946
T061UR	33002	1153	503	100.00%	56.46%	SAMEA5043011
T062UR	64983	10519	6302	100.00%	52.19%	SAMEA5042933
T063UR	53347	12793	4023	100.00%	40.00%	SAMEA5043041
T064UR	68122	23665	21094	100.00%	79.99%	SAMEA5042934
T065UR	51210	17070	1242	100.00%	65.30%	SAMEA5042977
T066UR	64589	26532	1911	100.00%	56.04%	SAMEA5042968
T067UR	67938	16248	4974	100.00%	50.12%	SAMEA5043005
T068UR	70192	28890	3698	100.00%	40.37%	SAMEA5043013
T069UR	60564	21236	17683	100.00%	43.97%	SAMEA5042957
T070UR	83453	20755	5034	100.00%	44.18%	SAMEA5043038
T071UR	80077	36770	29224	100.00%	97.68%	SAMEA5043007
T072UR	73469	29671	19787	100.00%	87.68%	SAMEA5042992
T073UR	73167	17577	3914	100.00%	54.09%	SAMEA5042989
T074UR	59084	23906	21347	100.00%	89.11%	SAMEA5042973
T075UR	60263	17726	15250	100.00%	70.37%	SAMEA5042976
T076UR	37428	809	514	100.00%	63.42%	SAMEA5042960
T078UR	76834	17034	4220	100.00%	66.75%	SAMEA5042958
T080UR	12172	609	61	100.00%	52.46%	SAMEA5042932
T081UR	63432	14841	6915	100.00%	86.49%	SAMEA5043029
T082UR	26941	693	609	100.00%	2.96%	SAMEA5043047
T083UR	69149	34307	30270	100.00%	95.45%	SAMEA5043055
T084UR	59304	25863	22866	100.00%	89.34%	SAMEA5042928
T085UR	65565	20426	1344	100.00%	78.57%	SAMEA5043030
T086UR	66605	23828	21243	100.00%	95.16%	SAMEA5043025
T087UR	62480	16656	6414	100.00%	76.55%	SAMEA5043057
T088UR	82733	32538	3794	100.00%	71.09%	SAMEA5042994
T089UR	110227	27223	11761	100.00%	24.49%	SAMEA5042944
T090UR	70526	29296	1917	100.00%	71.62%	SAMEA5043020
T091UR	27973	913	739	100.00%	27.74%	SAMEA5042952
T092UR	69694	10825	7894	100.00%	55.26%	SAMEA5043050
T093UR	58492	14656	7272	100.00%	84.27%	SAMEA5042980
T094UR	194564	59268	35224	100.00%	37.26%	SAMEA5042971
T095UR	42681	1009	560	100.00%	45.00%	SAMEA5042974

To explore the urinary microbiota in this dataset, morning midstream urine (UR) was self-collected prior to surgery from an exploratory cohort of 137 Chinese women recruited for the study (median age 31.6, range 22–48). As with our previous vagino-uterine microbiota study [2], all volunteers had conditions that were not known to involve infections [8]. From 95 women in the cohort, six locations within the female reproductive tract, including the lower third of the vagina (CL), the posterior fornix (CU), cervical mucus (CV), endometrium (ET), left and right fallopian tubes (FLL and FRL), and peritoneal fluid (PF) were also sampled. Their vagino-uterine microbiota information have been published previously [2]. After 16S rRNA gene amplicon sequencing, the sequencing reads were pre-processed for quality control and filtering, then clustered into OTUs (Methods, Table 1 and **OTU_table_urine.biom.hdf5** [8]).

**Figure 2.**
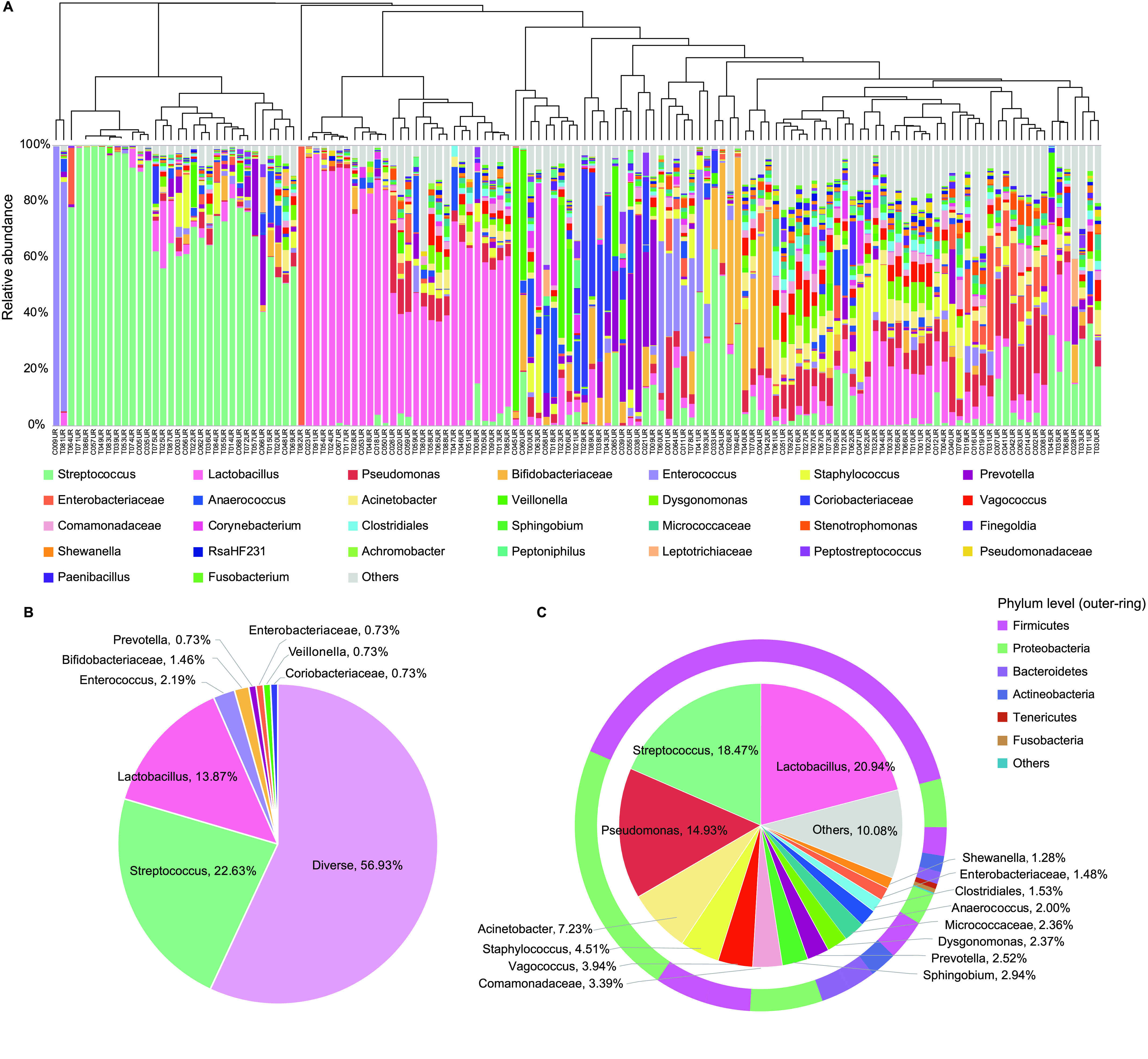
Urinary microbiota of the initial cohort of 137 Chinese reproductive-age women. (A) The relative abundances of genera detected in each individual are shown in the bar chart. The dendrogram is a result of a centroid linkage hierarchical clustering based on Euclidean distances between the microbial composition proportion of urinary bacterial communities. (B) The ratio of different urinary microbiota types. The genus whose relative abundance accounted for >50% in an individual was selected as an identified type. The genera that accounted for <50% of the microbiota in an individual were identified as diverse type. (C) Pie chart for the urinary microbial genera according to their median relative abundance. Genera that took up less than 1% of the microbiota are labelled together as ‘others’. The outer ring indicates the distribution of microbiota at the phylum level.

Due to anatomical structures, voided urine samples from women were considered to be easily contaminated by microbiota from the surrounding vulvovaginal region [22]. Most vaginal communities (88%) in this cohort were dominated by one genus with >50% relative abundance within data from individuals. In contrast, the urinary microbiota in this study showed more heterogeneity. 56.93% of the cohort harboured a diverse type represented significantly by bacteria, including *Streptococcus*, *Lactobacillus*, *Pseudomonas*, *Staphylococcus*, *Acinetobacter,* and *Vagococcus*, though none of these species were dominant, i.e. reached >50% relative abundance (Figure 2). In addition, 22.63% of the women harboured >50% *Streptococcus*, and 13.87% of the women harboured >50% *Lactobacillus* (Figure 2A, B). Rare subtypes such as *Enterococcus* (2.19%), *Bifidobacteriaceae* (1.46%), *Prevotella* (0.73%), *Enterobacteriaceae* (0.73%), *Coriobacteriaceae* (0.73%), and *Veillonella* (0.73%) were also detected in this cohort (Figure 2A, B). Notably, the median relative abundances of *Lactobacillus*, *Pseudomonas*, and *Acinetobacter* in the urine samples were more similar to the uterus samples (Figure 2C) [2]. At the phylum level, urinary microbiota were dominated by Firmicutes and Proteobacteria (Figure 2C).

### Cultivation of live bacteria from transurethral catheterized urine

The question of whether bacterial DNA signals have originated from live bacteria or fragments in the urine samples has been a subject of much debate [22]. To demonstrate the utility of the data for addressing this question, we performed a validation study using live bacteria cultures from urine samples provided by an additional cohort of 10 women.

We tried to culture and isolate bacterial colonies from freshly collected urine samples. Urine samples were serial diluted and spread on three different kinds of agar plates and incubated under both aerobic and anaerobic conditions. Six different positive isolates belonging to 5 genera, including *Lactobacillus*, *Staphylococcus*, *Clostridium*, *Enterococcus*, and *Propionibacterium* were obtained from 3 out of 10 subjects (Table 2). The 5 genera were also found as dominant in our 16S rRNA gene amplicon sequencing data and consistent with previous cultivation results of published papers [23–26] (Table 2). Reassuringly, no isolates were detected from the negative controls (sterile saline and ultrapure water). Therefore, these data verified the existence of live bacteria in the urine by obtaining isolates using conventional culturing methods.

**Table 2:** Identification of cultured microbial isolates from urine of the 10 additional women by sequencing of partial 16S rRNA gene.

Sample ID	Condition	Medium	16S rRNA gene-PCR Identification	Accessions	Identity (%)	Supported by previous cultivation
S001U	Anaerobic, 37 ^°^C	EG	*Clostridium cochlearium*	LR761333.1	99.26	Meijer-Severs *et al.* [24]
S001U	Anaerobic, 37 ^°^C	104	*Streptococcus sp. (S. tigurinus/S. mitis)*	LR761334.1	99.72	Hilt *et al.* [23]
S003U	Anaerobic, 37 ^°^C	BHI	*Enterococcus faecalis*	LR761335.1	99.91	Hilt *et al.* [23], Guzmàn *et al.* [25], Fraimow *et al.* [26],
S003U	Anaerobic, 37 ^°^C	104	*Lactobacillus crispatus*	LR761337.1	99.82	Hilt *et al.* [23]
S003U	Anaerobic, 37 ^°^C	104	*Propionibacterium granulosum*	LR761336.1	99.02	Ormerod *et al.* [27]
S008U	Anaerobic, 37 ^°^C	104, BHI, EG	*Streptococcus agalactiae*	LR761340.1, LR761339.1, LR761338.1	99.65, 99.35, 99.52	Hilt *et al.* [23]

### Considerable bacterial biomass revealed by qPCR

**Figure 3.**
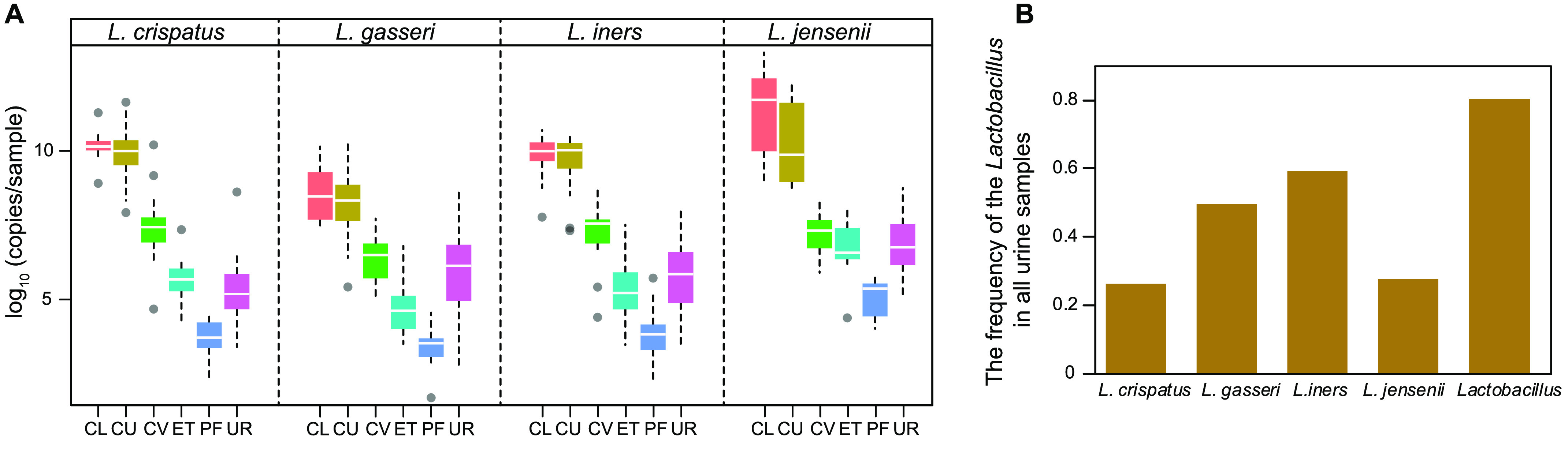
The concentrations of the dominant *Lactobacillus* species at urine and the reproductive tract. Samples derive from the initial cohort of 137 Chinese reproductive-age women. (A) The abundance of *L. iners*, *L. jensenii*, *L. crispatus* and *L. gasseri* calculated by qPCR results in different samples. Boxes denote the interquartile range (IQR) between the first and third quartiles (25th and 75th percentiles, respectively), and the lines inside the boxes denote the median. The whiskers denote the lowest and highest values within 1.5 times the IQR from the first and third quartiles, respectively. (B) The frequency of the respective *Lactobacillus* detected in all urine sample.

To provide additional evidence of the bacterial communities in the urine, a species-specific quantitative real-time PCR method was utilized to focus on the four common vaginal *Lactobacillus* species, i.e. *L. crispatus*, *L. iners*, *L. jensenii* and *L. gasseri* (**QPCR Lactobacillus.csv** [8]). The *Lactobacillus* species we examined presented a similar distribution and abundance along the female reproductive tract, and the corresponding urinary *Lactobacillus* ranged between the upper and lower reproductive tracts (Figure 3A). Among them, *L. iners* occurred most frequently (59%) in the urine samples, while *L. crispatus* only occurred in 26% of women sampled (Figure 3B). *L. iners* was reported far less protective against bacterial and viral infections compared to *L. crispatus* [28]. 80% of the cohort was detected to harbour at least one of these four *Lactobacillus* species (Figure 3B). The occurrence rate of *Lactobacillus* in the genus level of 16S rRNA gene amplicon sequencing data was 94% (Figure 2A). The total bacterial biomass is approximated by the ratio of the copy number from the result of qPCR to the relative abundance according to the result of 16S rRNA gene sequencing of the same sample (**QPCR bacterial_biomass.csv** [8]). The result gave an estimation of 10^7^ copies/sample, placing the urinary bacterial biomass between the vaginal-cervical sites (10^10^–10^11^ copies/sample) and the endometrium (ET) samples (10^6^–10^7^ copies/sample) [2] (Figure 3A), all of which were orders of magnitude above potential background noise [29]. These results were interestingly consistent with a weakly acidic pH of the urine, in comparison to pH < 4.5 in the vagina or pH ∼ 8 in the peritoneal fluid [30].

### Intra-individual similarity in the urine-reproductive tract microbiota

**Figure 4.**
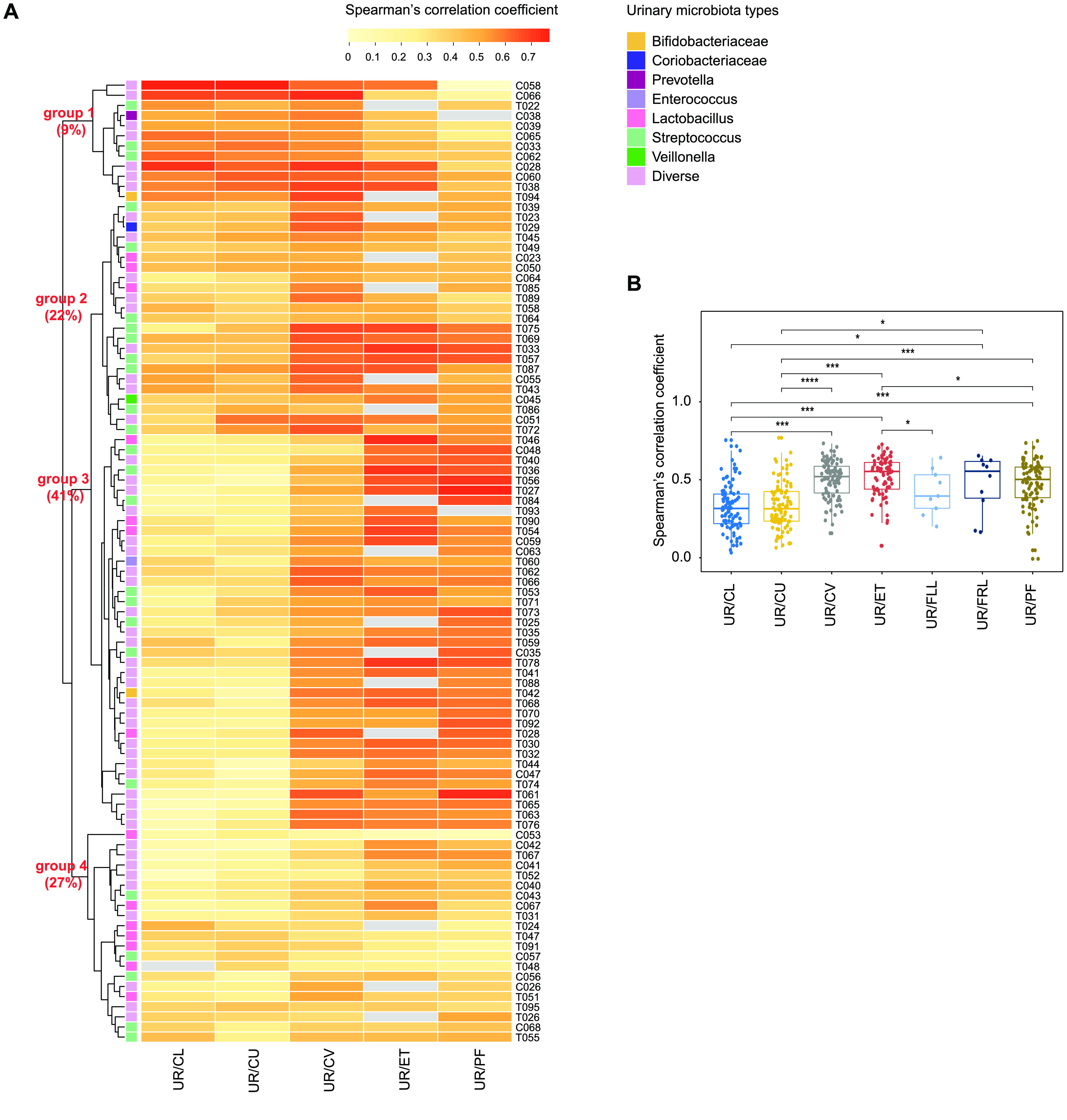
Similarity of the urine-reproductive tract microbiota within individuals. (A) Heatmap for the intra-individual Spearman’s correlation coefficient between microbiota identified in the urine and at different sites in the reproductive tract (**relative_abundance_correlation.csv** [8]). Samples derived from the initial cohort of 95 Chinese reproductive-age women, who collected both the urine and reproductive tract samples. As the number of samples from fallopian tubes (FLL, FRL) is too small, the correlation between microbiota in the urine and those in fallopian tubes are not shown. The dendrogram is a result of a centroid linkage hierarchical clustering based on Euclidean distances between the intra-individual Spearman’s correlation coefficient of different body sites. The colored squares illustrate the subtypes found within the urinary microbiome. (B) Spearman’s correlation coefficient between microbiota found in the urine and those from different sites of the reproductive tract. The Wilcoxon ranked sum test was used to calculate the difference. Boxes denote the interquartile range (IQR) between the first and third quartiles (25th and 75th percentiles, respectively), and the line inside the boxes denote the median. The whiskers denote the lowest and highest values within 1.5 times the IQR from the first and third quartiles, respectively. An asterisk denotes *p* <0.05, two asterisks denote *p* <0.01, three asterisks denote *p* <0.001.

**Figure 5.**
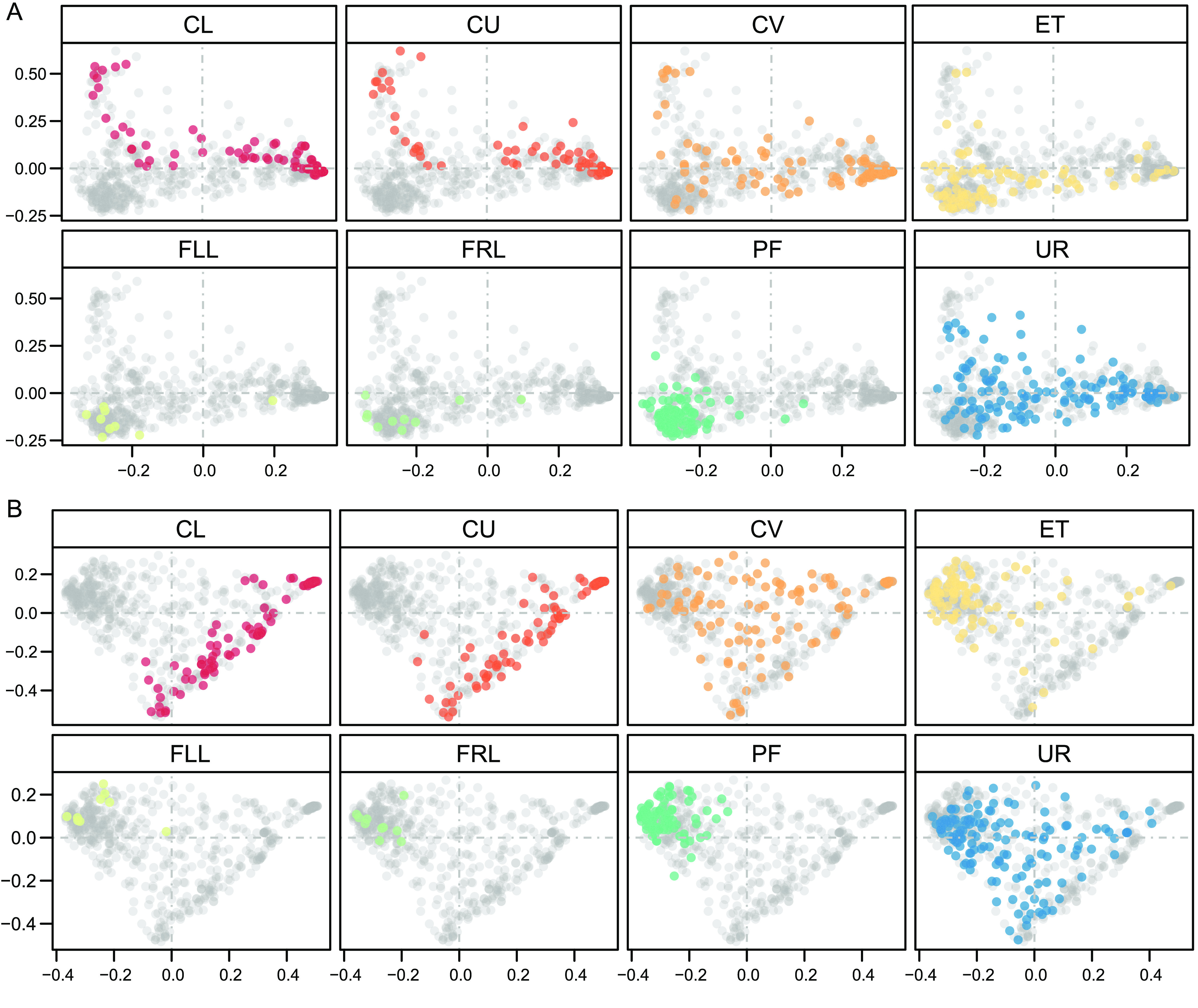
PCoA on the samples based on Unweighted-UniFrac (A) and Weighted-UniFrac (B) distances. Samples were taken from UR, CL, CU, and CV before operation, and from ET and PF during operation. Samples were derived from the initial cohort of 137 Chinese reproductive-age women. Each dot represents one sample (*n* =94 CL, 95 CU, 95 CV, 80 ET, 93 PF, 9 FLL, 10 FRL, and 137 UR).

To further assess the microbiota relationship between the urine and the six positions of the female reproductive tract, we computed intra-individual correlation between the microbial profiles in the urine and those found in different sites of the reproductive tract, and then clustered the individuals into 4 groups (Spearman’s correlation coefficient, Figure 4A, **relative_abundance_correlation.csv** [8]). Interestingly, the microbiota of group 3, which accounted for 41% of the cohort, showed significant correlation between the urine samples and the female reproductive tract samples, of which the coefficient increased gradually along the anatomical site from CL to CV, ET, and PF (Figure 4B). In contrast, 9% of women in group 1 presented a reverse trend. In group 2 (22%) and group 4 (27%), there appeared to be a weak relationship between the microbiota of the urine and female reproductive tract. Taken together, we observed the most similar distribution of microbiota between urine and CV/ET (Figure 4A). The principal coordinate analyses (PCoA) of the weighted and unweighted intra-individual UniFrac distance further corroborated our conclusion that there is an intra-individual similarity of the microbiota between the urine and the upper sites of female reproductive tract, especially the junction sites (CV and ET) (Figure 5).

### Lifestyle and clinical factors influencing the urinary microbiota

The human microbiome is dynamic and highly affected by its host environment. Age, menstrual cycle, benign conditions such as adenomyosis, and infertility due to endometriosis have previously been reported to shape the vagino-uterine microbiota [2]. With our comprehensive collection of demographic and baseline clinical characteristics from women of reproductive age (**sample_metadata.csv** [8]), such variations in the urinary microbiota can be explored in this dataset. Urinary microbial composition was significantly associated with these factors, such as age, surgical history, abortion, vaginal deliveries, experience of given birth (multipara vs. nullipara), infertility due to endometriosis, and hysteromyoma (PERMANOVA, *P* <0.05, *q* <0.05, Table 3). Although the urinary microbiota also correlated with some other factors, such as menstrual phase, contraception, endometriosis, pelvic adhesiolysis, and anemia, statistical significance was not achieved after controlling for multiple testing (PERMANOVA, *P* <0.05 but *q* >0.05, Table 3). The initial results here indicate a close link between the urinary microbiota and the general and diseased physiological conditions, and this link could be further understood by exploring this data more deeply.

**Table 3:** PERMANOVA for the influence of phenotypes on the urinary microbiota.

Phenotype	Bray-Curtis			Unweighted-UniFrac			Weighted-UniFrac		
	R2	*P* value	Fdr	R2	*P* value	Fdr	R2	*P* value	Fdr
Age	0.018	0.005	0.049	0.010	0.178	0.541	0.019	0.026	0.419
Age-2 groups	0.013	0.050	0.236	0.011	0.116	0.429	0.016	0.042	0.452
Age-3 groups	0.032	0.026	0.150	0.025	0.228	0.577	0.030	0.135	0.539
Pulses	0.015	0.019	0.131	0.010	0.159	0.505	0.015	0.072	0.456
Frequent colds	0.011	0.080	0.270	0.011	0.108	0.429	0.021	0.017	0.419
Antibiotics	0.014	0.036	0.194	0.012	0.108	0.429	0.007	0.525	0.782
Constipation	0.011	0.114	0.325	0.014	0.039	0.400	0.018	0.033	0.419
Surgical history	0.018	0.005	0.049	0.018	0.006	0.172	0.034	0.001	0.091
Abdominal surgical history	0.010	0.187	0.418	0.007	0.466	0.755	0.019	0.030	0.419
Menstrual cycle	0.009	0.200	0.421	0.018	0.005	0.172	0.015	0.065	0.455
Menstrual phase (lower)	0.018	0.260	0.468	0.024	0.048	0.408	0.020	0.207	0.623
Menstrual phase (upper)	0.018	0.006	0.056	0.018	0.009	0.172	0.014	0.096	0.456
Contraception	0.044	0.010	0.086	0.038	0.098	0.429	0.029	0.470	0.782
Vaginal deliveries	0.018	0.003	0.049	0.016	0.014	0.172	0.016	0.051	0.455
Abortion	0.028	0.004	0.049	0.014	0.239	0.585	0.016	0.198	0.623
Multipara / nullipara	0.019	0.003	0.049	0.017	0.014	0.172	0.013	0.091	0.456
Infertility due to endometriosis	0.045	0.000	0.008	0.029	0.013	0.172	0.019	0.181	0.599
Endometriosis	0.014	0.022	0.141	0.011	0.095	0.429	0.005	0.644	0.857
Pelvic adhesiolysis	0.008	0.346	0.572	0.013	0.042	0.400	0.006	0.489	0.782
Anemia	0.016	0.012	0.090	0.008	0.354	0.740	0.006	0.511	0.782
Hysteromyoma	0.018	0.003	0.049	0.012	0.057	0.429	0.021	0.014	0.419

### Potential uses

As a large-scale cohort for studying the female urinary microbiota, our data provide a useful baseline and reference dataset in women of reproductive age. We also explored the association between the composition of urinary microbiota and that of the female reproductive tract microbiota. It is valuable to note that a higher intra-individual compositional similarity was observed between the microbiota of the urine and those of the cervical canal/uterus than between the microbiota of the urine and those of the vagina. This finding indicates that sampling of midstream urine (the least invasive and the easiest way) could be potentially used to survey the micro-environment of the cervical canal and uterus in the general population. This is relevant to the demonstrated associations between the urinary microbiota and various uterine-related diseases, such as hysteromyoma and infertility due to endometriosis. Our data provide a reference for clinical diagnosis and warrants further detailed exploration.

There are three limitations for this study. Firstly, as it was not possible to directly sample the upper reproductive tract of perfectly healthy women, we have included women who underwent minimally invasive laparoscopy or laparotomy for conditions that are not known to involve infection. This was the best proxy for sampling the upper reproductive tract in healthy women. Nevertheless, the relevance of the urinary microbiota between healthy women and women in our cohort would require further comparison. Secondly, for the low bacterial biomass of urine samples, a more comprehensive sampling process should be taken into consideration in subsequent studies, such as disinfection of the urethra and vulvovaginal region with 75% alcohol before urine self-collection, including a sample of sterile saline with the self-collection kit as a negative control and asking participants to fill another vial with it immediately following urine collection. A comparison of the microbial composition between the catheter-collected and self-collected specimens in the same individual would also require further inspection. Together, we hope that this dataset helps promote a new round of accelerated discoveries, including a novel scientific explanation for uterine-related diseases via longitudinal studies on the microbiota of the urinary and reproductive tracts.

## Data Availability

The sequence reads generated by 16S rRNA gene amplicon sequencing have been deposited in both the European Nucleotide Archive with the accession number PRJEB29341 and the CNSA (https://db.cngb.org/cnsa/) of CNGB database with accession code CNP0000166. Additional data, result and a STORMS (Strengthening The Organizing and Reporting of Microbiome Studies) checklist are available from the *GigaScience* GigaDB repository [8]. The sequences of bacterial isolates have been deposited in the European Nucleotide Archive with the accession number PRJEB36743.
